# Layer-by-layer assembled film can serve as an enhanced reaction environment for Diels–Alder reaction

**DOI:** 10.3389/fchem.2024.1524096

**Published:** 2024-12-12

**Authors:** Nanami Fujisawa, Mitsuhiro Ebara

**Affiliations:** ^1^ Research Center for Macromolecules and Biomaterials, National Institute for Materials Science (NIMS), Tsukuba, Japan; ^2^ Graduate School of Pure and Applied Sciences, University of Tsukuba, Tsukuba, Japan; ^3^ Department of Materials Science and Technology, Tokyo University of Science, Tokyo, Japan

**Keywords:** Diels-Alder reaction, layer-by-layer, quartz crystal microbalance, magnetic nanoparticles, AC magnetic field

## Abstract

Although the Diels–Alder reaction (DA) has garnered significant attention due to its numerous advantages, its long reaction time is a drawback. Herein, we investigated the effects of polarity difference on DA using Layer-by-Layer (LbL) films comprising polycationic polyallylamine hydrochloride and polyanionic poly (styrenesulfonic acid-co-furfuryl methacrylate) [poly (SS-*co*-FMA)] as the reaction environment. First, furan composition in poly (SS-*co*-FMA) was adjusted to be 19 mol% to achieve good water solubility and layer deposition. The successful formation of LbL films with 8 and 40 layers was confirmed by quartz crystal microbalance. The polarity within films and, consequently, the DA efficiency between furfuryl methacrylate and the maleimide in MAL-PEG_2_-NHS increased with an increasing number of layers up to 40 layers without requiring chemical modification on the reaction site of DA or any catalysts. Furthermore, we employed the LbL coating on the surface of magnetic nanoparticles (MNPs). The retro DA reaction (rDA) was successfully triggered by heating the MNPs by AC magnetic field. We believe that the proposed technology can serve as an enhanced DA reaction environment as well as temporal/spatial control of rDA in various applications.

## 1 Introduction

Dynamic covalent bonds are chemical bonds that reversibly form under equilibrium-controlled conditions. These bonds first reach transition states upon being subjected to external stimuli such as heating or catalysis, following which they achieve stable states that are not their initial states. The Diels–Alder (DA) reaction, which is highly direct and does not require the use of a catalyst, (Rowan et al., 2002; Maeda et al., 2009; Gregoritza and Brandl, 2015), is based on the chemically selective [4 + 2] cyclization between the electron-withdrawing group of a diene and the electron-donating group of a dienophile; it is particularly important in the formation of ring structures and is applied for the production of compounds such as small-molecule pharmaceuticals (Nicolaou et al., 2002). However, the reaction time for DA reactions can be relatively long, which can hinder practical application; in our previous study, a DA reaction required 120 h to reach completion (Fujisawa et al., 2021). The reaction time can be controlled by changing the energy state of the reaction site via chemical modification (Froidevaux et al., 2015). In addition, reaction times are highly dependent on the external environment, (Kiselev and Konovalov, 2009), including the nature of the reaction solvent (e.g., solvent polarity, hydrogen bond formation with reactants, and the use of ionic liquids), the presence of catalysts, the formation of reaction environments by molecular aggregates (Itami et al., 2002), and the reaction temperature. From this background, Baker et al., has recently proposed to use an enzyme as a reaction filed which can catalyze the DA reaction (Siegel et al., 2010). They described the *de novo* computational design and experimental characterization of enzymes catalyzing a bimolecular DA reaction with high stereoselectivity and substrate specificity.

In general, the progression of an endothermic reactions was accelerated with an increase in temperature; however, in the case of a DA reaction, when the energetic threshold is exceeded, the revere reaction, so called retro DA reaction (rDA), proceeds backward to form the original structure (Gregoritza and Brandl, 2015). Also of particular note in the DA reaction is the fact that this chemo selective reaction can proceed in both organic solvents and water requiring no catalyst. Therefore, many applications of DA/rDA reaction in the biomedical fields have been reported by taking advantages of the thermal reversibility and biocompatibility (e,.g., controlled drug release technologies, etc.). On the other hand, the major limitation is its relatively high working temperature. For example, we have previously shown that the rDA reaction occurred at 90°C, which is too high for living organisms (Fujisawa et al., 2021). From these perspectives, we consider that discovering and establishing an efficient DA reaction environment are significantly important to overcome current limitations.

**FIGURE 1:**
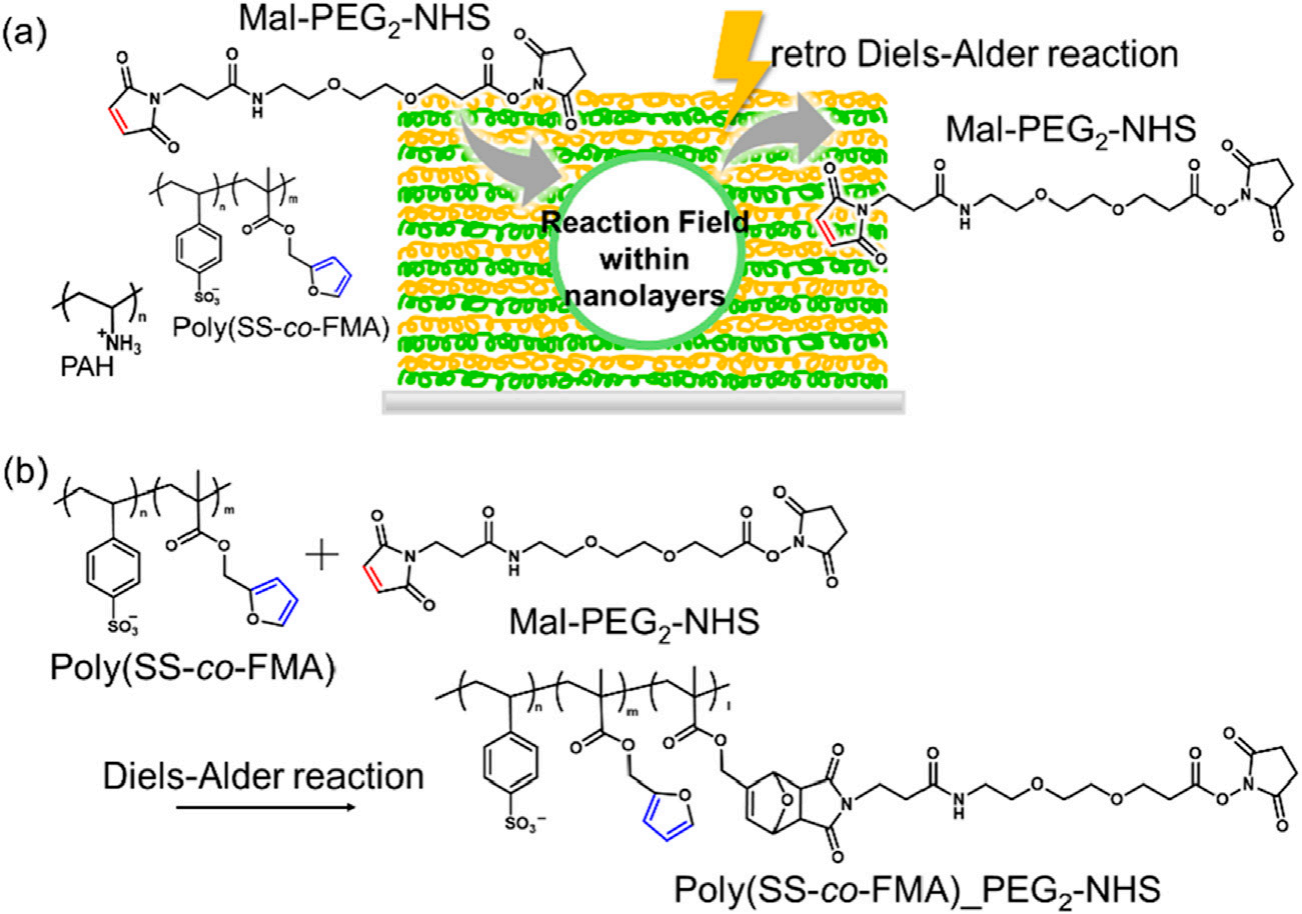
**(A)** Layer-by-Layer assembled reaction environment comprising PAH and poly (SS-*co*-FMA). **(B)** Diels–Alder reaction between poly (SS-*co*-FMA) and MAL-PEG_2_-NHS. Styrene sulfonic acid sodium salt; SS, furfuryl methacrylate; FMA, polyallylamine hydrochloride; PAH.

In this study, we aimed to investigate the effects of the polarity of the reaction environment on DA reactions through a novel approach. As mentioned previously, the reaction rate of a DA reaction can be controlled without changing the chemical structure of the reaction site but its polarity. For instance, Jung et al. measured the reaction rates of DA reactions in various solvents with different polarities and found them to be accelerated in highly polar solvents such as DMSO (Jung and Gervay, 1989). Notably, we used the Layer-by-Layer (LbL) self-assembly technology instead of solvents to alter the external environment (Figure 1A). LbL has been widely used to fabricate nanometer-scale multilayered membranes and has been adapted to sequentially adsorb oppositely charged polyelectrolytes (PEs) onto substrates to fabricate membranes comprising a variety of polymer species (Caruso et al., 2001; Ariga et al., 2006; Ariga et al., 2019). Interestingly, Tedeschi et al. reported that LbL layers are highly polar; (Tedeschi et al., 2001); they prepared LbL layers of various polyanions to form the polycation polyallylamine hydrochloride (PAH) and showed that the difference in internal polarity can be measured as an indicator of the difference in pyrene fluorescence (Bains et al., 2011). Though LbL has been used for various applications, there have been no reports on techniques that utilize the internal polarity of the layers. Herein, we show that the use of LbL layers can replace chemical modification to promote DA reactions and potentially increase their reaction rates. We evaluated the polarities of the LbL membranes using pyrene and, correspondingly, the amounts of MAL-PEG_2_-NHS bound in the DA reaction. In addition, we applied the LbL coating on the surface of magnetic nanoparticles (MNPs). Because the MNPs can generate heat by irradiating AC magnetic field, the progression of reverse reaction (retro DA reaction) was also evaluated.

## 2 Results and discussion

### 2.1 Synthesis of poly (SS-*co*-FMA)

**FIGURE 2:**
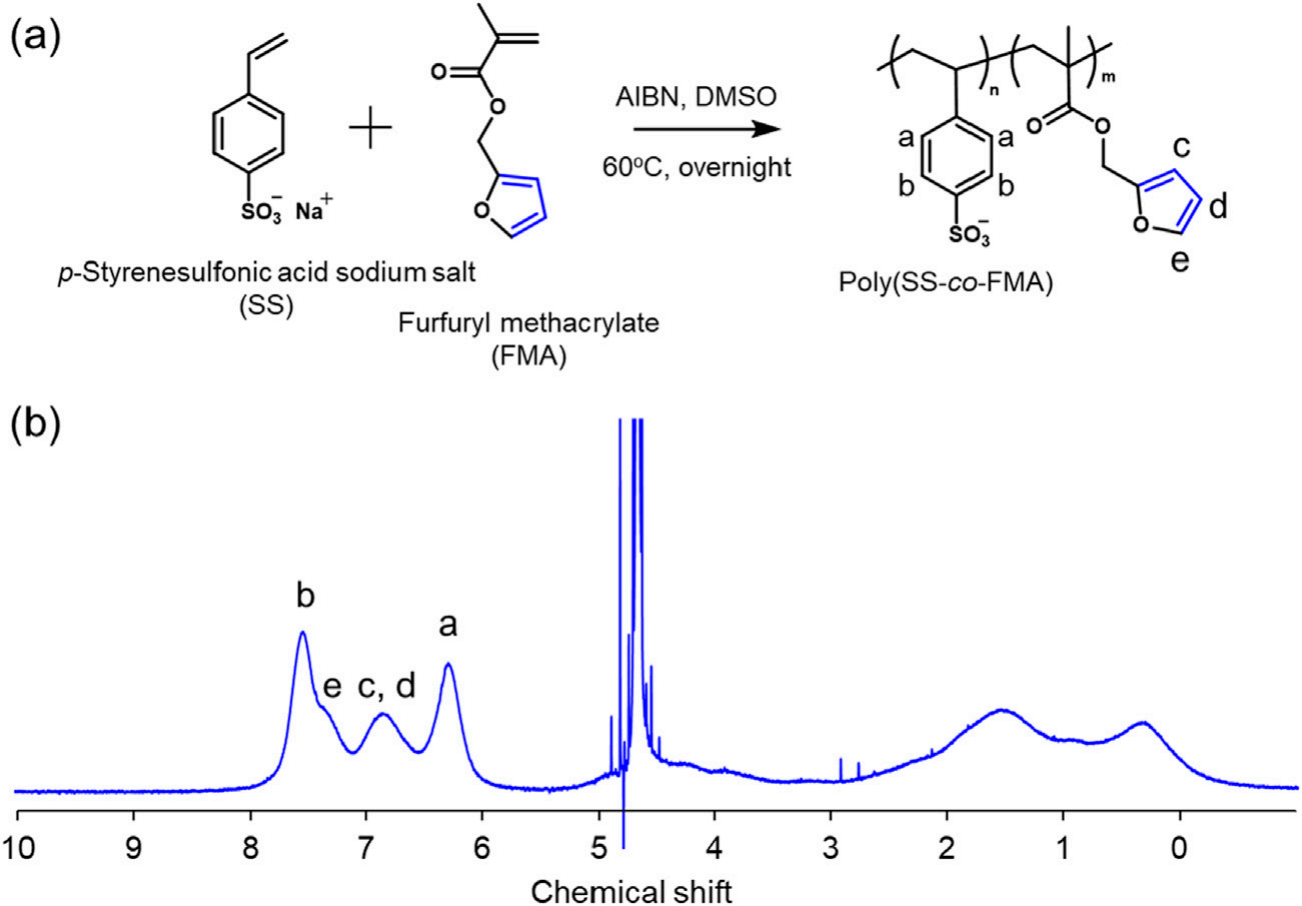
**(A)** Synthesis scheme of poly (SS-*co*-FMA) and **(B)** ^1^H NMR spectrum of poly (SS-*co*-FMA) in D_2_O by using 400 MHz NMR. The feed ratio of SS and FMA was 60 mol% and 40 mol%, respectively.

First, the free-radical polymerization of copolymers of furfuryl methacrylate (FMA) and styrene sulfonic acid sodium salt (SS) was performed at their respective proportions to provide reaction sites for the DA reaction. The reason for copolymerization with SS is that FMA must be dispersed in water owing to its hydrophobicity, but it does not have a charge for the electrostatic interactions in LbLs; therefore, it must be copolymerized with SS, an anion. The amount of FMA to be introduced was calculated using ^1^H nuclear magnetic resonance (^1^H NMR) spectroscopy and was found to be 19 mol% (Figure 2B). Gel permeation chromatography (GPC) revealed that the synthesized polymer, poly (styrenesulfonic acid-*co*-furfuryl methacrylate (poly (SS-*co*-FMA)), had a molecular weight of approximately 50k and a polydispersity index (PDI) of 2.9. To determine the composition of poly (SS-*co*-FMA), UV-vis spectra were recorded for a poly (SS-*co*-FMA) solution (Supplementary Figure S1). Absorption peaks were observed at 262 and 300–350 nm, corresponding to the phenyl and furan groups (Yılmaztürk et al., 2009; Liu et al., 2013), respectively (Supplementary Figure S1A); the phenyl absorption tended to decrease with increasing furan content.

### 2.2 Layer-by-layer thin film formation

**FIGURE 3:**
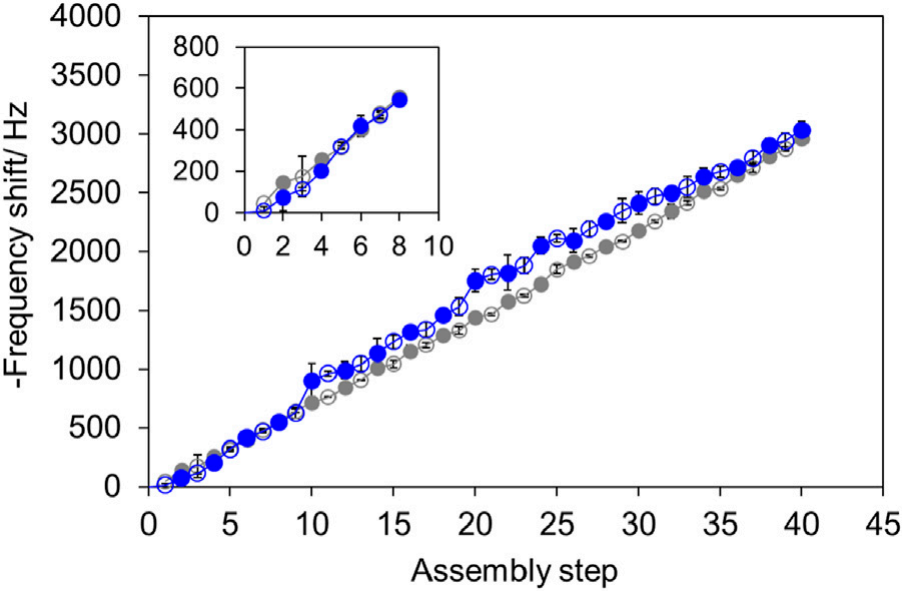
Adsorption of [PAH/PolySS] (gray) and [PAH/Poly (SS-*co*-FMA)] (blue). PAH (open), polySS and poly (SS-*co*-FMA) (closed).

Poly (SS-*co*-FMA) with 19 mol% of furan group, which was soluble in water, was used to evaluate the LbL process. Poly (SS-*co*-FMA) is entirely anionic; therefore, it was stacked with the cationic PAH via electrostatic interactions. Alternately stacked polymer layers were achieved by repeatedly immersing quartz substrates in alternating polymer solutions followed by washing and drying. The adsorbed polymers were evaluated using a quartz crystal microbalance (QCM) (Figure 3); the results showed that alternating stacking can be achieved even with furan-containing polymer. Up to 40 layers were evaluated; it was found that alternating stacking continued stably even for this high number of layers. We therefore concluded that DA reaction sites were successfully introduced into the LbL membrane.

### 2.3 Polarity in layer-by-layer film

**FIGURE 4:**
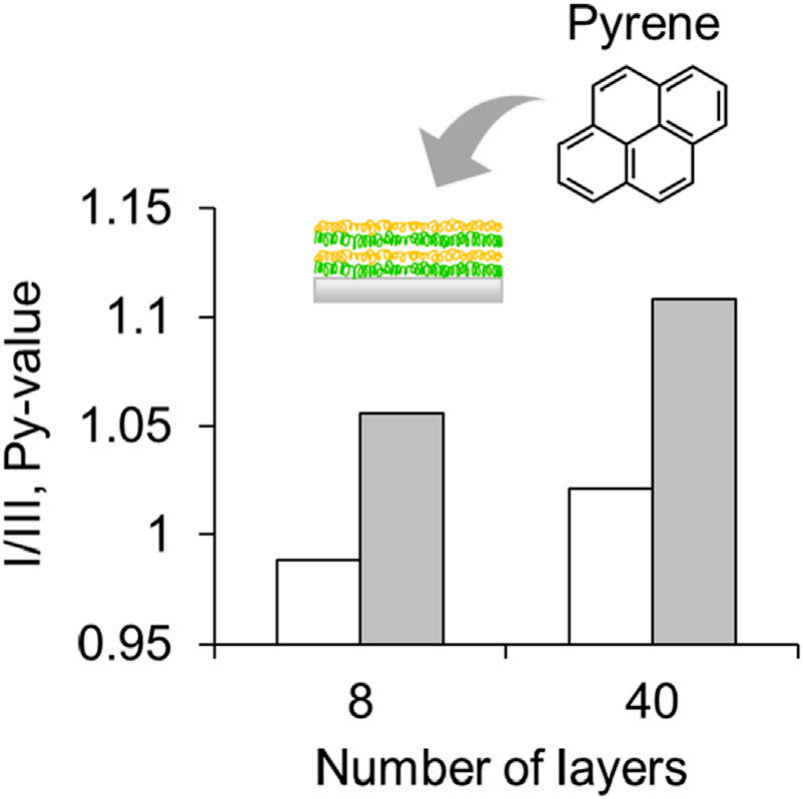
Fluorescence intensity ratio (I/III, Py-value) of pyrene in 8 and 40 layers of [PAH/Poly (SS-*co*-FMA)]. Dry state (open) and immersed in MiliQ water (closed).

To investigate the relative polarity changes within the LbL layers, the LbL layers stacked on the quartz slide surface were immersed in a 1.0 × 10^−4^ g L^–1^ pyrene solution; pyrene is a commonly used polarity-sensitive probe. The emission spectrum of the pyrene monomer showed five prominent fluorescence peaks between 370 and 400 nm. The first band (I) increased in intensity compared to the third band (III) when exposed to the polarity of the solvent owing to coupling between the electronic and vibrational states (Supplementary Figure S2). The first and third vibronic peaks ratio, I/III, is generally referred to as the Py value (Tedeschi et al., 2001), and indicates the polarity of the solvent. Here, an increase in Py value was observed for the 40 layers film compared to the 8 layers film, indicating an increase in polarity as the number of layers is increased (Figure 4). Generally, polarity within polymer layers is known to be influenced by their environments, such as dielectric constant. Because of the dense stacking layer structure of PAH/Poly (SS-*co*-FMA), the dielectric constant increases with an increase of layer numbers. The Py value of the dry film and the film immersed in MiliQ water are compared, and both show a similar trend of increased Py value, indicating that it is not the fluorescence of pyrene seeping from the film, but the fluorescence of pyrene loaded on the film.

### 2.4 Diels–Alder reaction in poly (SS-*co*-FMA) solution

**FIGURE 5:**
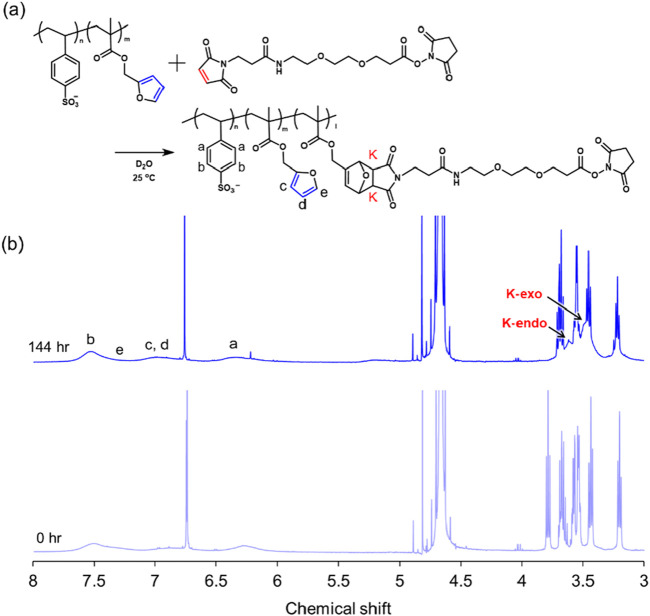
Scheme of the Diels–Alder reaction **(A)** and ^1^H NMR spectral changes after 144 h of Diels–Alder reaction **(B)**.

The DA reaction is widely used in small-molecule synthesis to form cyclic structures in molecules. Because the reaction proceeds in water or organic solvents and does not require a catalyst, it is applicable to biomaterials. Furan efficiently undergoes the DA reaction with maleimide because furan is an electron-donating group and maleimide is an electron-withdrawing group, and the pericyclic reaction proceeds efficiently because of the large energy gap of the highest occupied molecular orbital/lowest unoccupied molecular orbital. (Froidevaux et al., 2015). The progression of the DA reaction was confirmed by ^1^H NMR spectroscopy (Figure 5) and was evaluated according to the K peaks of the newly formed protons after cyclization, which were found to be stereoisomeric K-endo (3.60 ppm) and K-exo (3.49 ppm). The conversion rate of the DA reaction between furan and MAL-PEG_2_-NHS in the polymer increased with time, with saturation at approximately 50%–60% after 144 h. This rate depends strongly on the reaction environment, including the reaction solvent and temperature.

### 2.5 Diels–Alder reaction in poly (SS-*co*-FMA) layer-by-layer film

**FIGURE 6:**
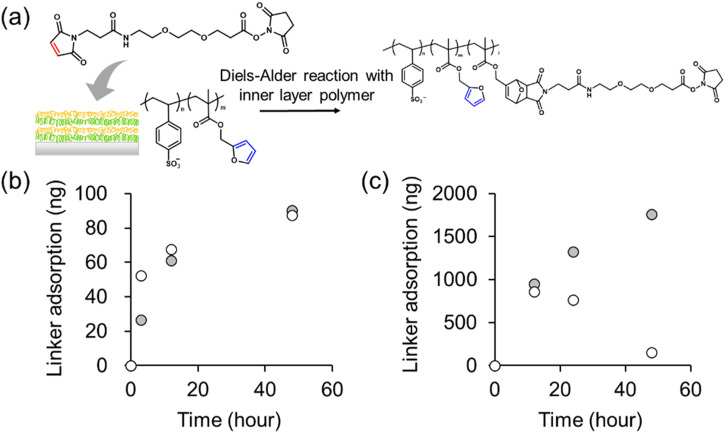
**(A)** DA reaction of LbL layers in MAL-PEG_2_-NHS solution. Adsorption of MAL-PEG_2_-NHS over time in **(B)** 8 layers and **(C)** 40 layers of [PAH/PolySS)] (open) and [PAH/Poly (SS-*co*-FMA)] (closed).

Finally, MAL-PEG_2_-NHS was permeated into the membranes with 8 and 40 layers, and the DA reaction was investigated. As shown in Figure 6A, 8 and 40 layers were stacked on quartz slides, immersed in a 1.0 mg mL^–1^ of MAL-PEG_2_-NHS solution, and the frequencies of the quartz substrates in the dry state were measured. The frequencies decreased as the weight of the material bonded to the crystals increased. The Sauerbrey’s equation was then used to calculate the actual amounts of the adsorbed substance by calculating the total weight of the bound material; the total weight of the introduced MAL-PEG_2_-NHS was plotted against the frequencies of the 8 and 40 layers samples. Figure 6B shows the weight change of the membranes stacked with 8 layers of the [PAH/Poly (SS-*co*-FMA)] film and 8 layers of a [PAH/PolySS] film. PolySS is a styrene sulfonic acid sodium salt homopolymer that does not contain furans. However, even with the [PAH/PolySS] film, a weight gain was observed. This may be attributed to the nonspecific interaction of a MAL-PEG_2_-NHS between the layers. Therefore, we evaluated 40 layers of [PAH/PolySS] and [PAH/Poly (SS-*co*-FMA)] to observe the differences in adsorption with and without furan (Figure 6C). Even with 40 layers of [PAH/PolySS], MAL-PEG_2_-NHS adsorption was observed after a reaction time of 12 h. However, the total weight of the 40 layers of [PAH/PolySS] decreased after 24 h, whereas that of the 40 layers of [PAH/Poly (SS-*co*-FMA)] continued to increase with reaction time. This is thought to be because in the 40 layers of [PAH/PolySS], the MAL-PEG_2_-NHS were not chemically bonded but only dispersed, and thus were released out of the layer by diffusion. In contrast, in the 40 layers of [PAH/Poly (SS-*co*-FMA)], the MAL-PEG_2_-NHS was chemically bound to FMA via the DA reaction, which is thought to have inhibited MAL-PEG_2_-NHS release. However, of the total weight of [PAH/Poly (SS-*co*-FMA)] after 48 h, 14,904 ng of the MAL-PEG_2_-NHS was adsorbed, which was 137 times more than the amount of furan groups in the 40 layers of [PAH/Poly (SS-*co*-FMA)]. Thus, it should be noted that this weight includes the MAL-PEG_2_-NHS dispersed between the layers. However, there is a difference in adsorption with respect to stacking.

### 2.6 MAL-PEG_2_-NHS release in response to AC magnetic field from layer-by-layer film

**FIGURE 7:**
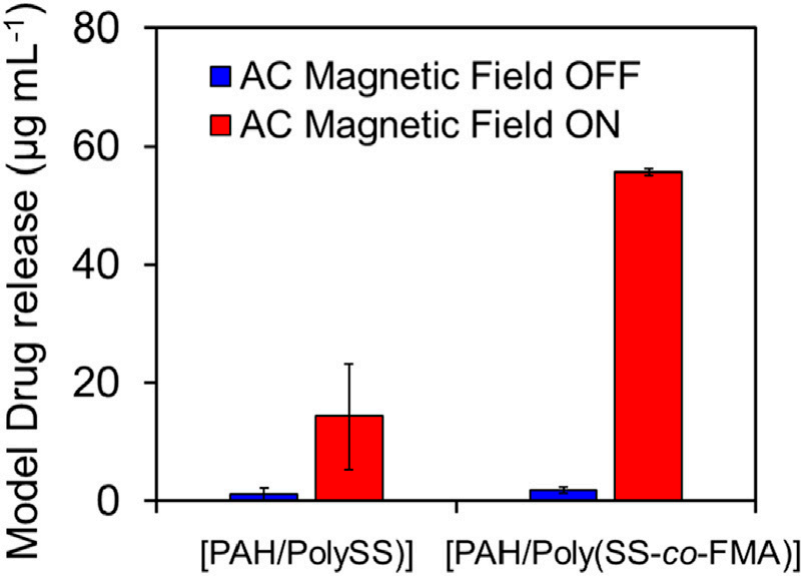
Release of MAL-PEG_2_-NHS from MNPs coated with 8 layers of [PAH/PolySS)] and [PAH/Poly (SS-*co*-FMA)] with (red) and without (blue) AC magnetic field irradiation for 15 min.

Finally, a MAL-PEG_2_-NHS was introduced to the polymer-coated MNPs via the Diels–Alder reaction. MNPs have been exploited as heating agents by taking advantage of heat generation by the irradiation of AC magnetic field. This enables local heating around MNPs. Because DA-based covalent bonds have been known to turn to its original form upon heating (called retro DA reaction; rDA), AC magnetic field irradiation can trigger the release of MAL-PEG_2_-NHS from MNPs via rDA reaction. The magnetic nanoparticles decorated with 8 layers of [PAH/Poly (SS-co-FMA)] were measured by dynamic light scattering (DLS) with a particle size of 271 nm and a PDI of 0.16. Figure 7 compares the released amount of MAL-PEG_2_-NHS from MNPs with 8 layers of [PAH/PolySS)] and [PAH/Poly (SS-*co*-FMA)] with/without AC magnetic field application for 15 min. As expected, the accelerated release of MAL-PEG_2_-NHS has been observed upon AC magnetic field application, while no or little release was observed without AC magnetic field. According to our previous study, heat generation from MNPs was calculated to be approximately 6.0 mJ mg^−1^ for 15 min and this energy is much larger than that required for the rDA (52.0 mJ mg^−1^). (Fujisawa et al., 2024). Therefore, rDA-based MAL-PEG2-NHS release was successfully achieved using LbL-coated MNPs. On the other hand, MAL-PEG_2_-NHS release was also observed even in [PAH/PolySS)], which does not contain furan groups. This is due to the release of the MAL-PEG_2_-NHS entrapped within the layers noncovalently. Although optimization of the proposed system is still required to achieve perfect ON-OFF MAL-PEG_2_-NHS release control, these results indicate a tremendous potential for facile regulation of rDA reaction in biomedical fields.

## 3 Experimental

### 3.1 Materials

*p*-Styrenesulfonic acid sodium salt (SS), dimethyl sulfoxide (DMSO), 2,2′-azobis (isobutyronitrile) (AIBN), 1 mol L^-1^ sodium chloride (NaCl) solution, pyrene, deuterium oxide (99.8%, for NMR), lithium chloride (LiCl), acetonitrile (ACN; high-performance liquid chromatography grade), sulfuric acid (H_2_SO_4_), and hydrogen peroxide (H_2_O_2_, 30 wt%) were purchased from FUJIFILM Wako Pure Chemical Corporation (Osaka, Japan). Maleimide-PEG_2_-NHS (MAL-PEG_2_-NHS, >98.0%) was purchased from Tokyo Chemical Industry Co., Ltd. (Tokyo, Japan). Ultrapure distilled Milli-Q water was used in this study (Merck, Darmstadt, Germany). Furfuryl methacrylate (FMA, 97%) containing 200 ppm monomethyl ether hydroquinone as an inhibitor) was removed by passing through an alumina oxide column before use. Polyallylamine hydrochloride (PAH; average molecular weight (M_w_) of 50k) was purchased from Sigma–Aldrich (MO, USA). An iron oxide nanoparticle water suspension and EMG 607 (cationic surfactant) were purchased from Ferrotec Material Technologies Corporation (CA, USA).

### 3.2 Synthesis of poly (SS-*co*-FMA)

Poly (SS-*co*-FMA) was polymerized via free-radical polymerization. Briefly, SS (4.0 g), FMA, and AIBN (0.065 mol% of the total monomer concentration) were dissolved in 11.0 mL of DMSO; the total amount of monomer was 19.4 mmol. All the reactants were placed in a 20 mL glass vial, mixed well, and sealed with a rubber cap. The reaction solution was purged with nitrogen gas for 20 min. The polymerization was conducted overnight at 60 °C, following which the polymer solution was loaded into Spectra/Por^®^ dialysis tubing with a M_w_ cutoff of 3.5 kDa (Repligen, MA, USA) against distilled water for 3 days. The dialyzed solutions were freeze-dried for 2 days, and the polymer was obtained as a white powder. The chemical structure of poly (SS-*co*-FMA) was confirmed by ^1^H NMR spectroscopy at 400 MHz (JEOL Ltd., Tokyo, Japan). All the NMR samples were prepared at a concentration of 10.0 mg mL^-1^ in D_2_O. The average molecular weight of the polymers was determined via gel permeation chromatography (GPC; GPC101, Shodex Corporation, Tokyo, Japan). The mobile phase had a flow rate of 0.8 mL min^-1^ at 40 °C and consisted of 0.5 wt% LiCl in ACN:H_2_O (4:6). A polystyrene standard was used to calculate the M_w_, M_n_ and PDI.

### 3.3 Characterization of I/III ratio in poly (SS-*co*-FMA)/PAH layers

An aqueous solution of pyrene was prepared to a concentration of 1.0 × 10^−7^ g mL^-1^. Because pyrene is difficult to dissolve in water at high concentrations, 49.0 mg of pyrene was first weighed and mixed up with 100 mL of acetone using a measuring flask (4.9 × 10^−4^ g mL^-1^). Then, 204 µL was taken from the prepared pyrene solution and was metered up with 100 mL of MiliQ water using a volumetric flask (1.0 × 10^−7^ g mL^-1^). Layers of 8 and 40 films were prepared on the quartz slides as described above, and then immersed in a pyrene solution for 3 days to incorporate pyrene into the polymer layers. The crystal slides were removed from the pyrene solution, washed three times with MiliQ water, and placed in a cuvette filled with and without MiliQ water by fluorescence intensity measurement.

### 3.4 Characterization of PAH/poly (SS-*co*-FMA) assembly by QCM

Poly (SS-*co*-FMA) and PAH solutions were prepared in advance at concentrations of 5.0 mg mL^-1^ in Milli-Q water and 150 mM NaCl. Before the nanolayer formation on quartz crystal substrate, piranha solutions were prepared in H_2_SO_4_:H_2_O_2_ (30 wt% in water) = 3:1 v/v% with ice cooling and then dropped with a Pasteur pipette onto the quartz crystal slides for 1 min. Subsequently, the electrodes were rinsed with Milli-Q water and dried with nitrogen gas to clean the electrode surface via blasting. Prior to the formation of [PAH/Poly (SS-*co*-FMA)] assembly on the gold-coated quartz crystal substrate (QA-A9M-AU(SEP), SEIKO, E.G.,&G Co., Ltd., Tokyo, Japan), the QCM leads were protected with silicone rubber gel (KE-1830, Shin-Etsu Chemical Co., Ltd., Tokyo, Japan) to prevent degradation during immersion in the polymer solutions. Piranha solutions were dropped with a Pasteur pipette onto the QCM electrodes for 1 min. Subsequently, the electrodes were rinsed with Milli-Q water and dried with nitrogen gas to clean the electrode surface via blasting. The cleaned QCM electrodes were immersed in a 5.0 mgmL^-1^ PAH aqueous solution for 5 min, removed, washed thoroughly with Milli-Q water, and dried with nitrogen gas. The frequencies of the QCM electrodes were then measured using a QCA917 device (SEIKO, E.G.,&G Co., Ltd., Tokyo, Japan). Next, the QCM electrodes were immersed again in a 5.0 mg mL^-1^ of poly (SS-*co*-FMA) aqueous solution for 5 min, removed, washed, and dried, and their frequencies were measured again. This process was repeated eight times, and the reduction in the frequency of each polymer layer was plotted. Error bars represent standard deviations for n > 6 of QCM measurements.

### 3.5 Characterization of Diels–Alder reaction in poly (SS-*co*-FMA) solution

MAL-PEG_2_-NHS (161.0 mg) was dissolved in 11.5 mL of D_2_O. Poly (SS-*co*-FMA19) (100.0 mg) was placed in the glass vial. An MAL-PEG_2_-NHS solution (1.0 mL) was added to the polymer-containing in the glass vial and allowed to react with stirring. The reaction solution (0.7 mL) was collected at various time points (0 and 144 h) and directly analyzed via ^1^H NMR spectroscopy. The progress of the DA reaction corresponded to K-endo (3.60 ppm) and K-exo (3.49 ppm), which appeared after the reaction.

### 3.6 Characterization of Diels–Alder reaction in PAH/poly (SS-*co*-FMA) nanolayers by QCM

MAL-PEG_2_-NHS solution was prepared in Mili-Q water to 1.0 mg mL^-1^. Quartz slides with stacked polymer layers were immersed and the frequency of the quartz crystal was measured at each time (3–48 h). Here, the mass of MAL-PEG_2_-NHS adsorbed ( ∆m ) were calculated from the decrease in frequency using Sauerbrey’s equation (Vogt et al., 2004; Uto et al., 2008). The density of the quartz crystal ( $\rho_{q}$ ) was 2.648 g cm^-3^, the modulus of elasticity of the crystal ( $\mu_{q}$ ) was 2.947 × 10^−13^ g m^-1^ s^-2^, and the standard resonance frequency of the crystal ( $f_{0}$ ) was calculated from the data of QA-A9M-AU (SEP) as 8.95 MHz and the electrode diameter of the gold substrate of the quartz crystal ( A ) as 5 mm φ.

$$-∆f=\frac{2{f_{0}}^{2}}{A\sqrt{\rho_{q}\mu_{q}}}\times ∆m$$

### 3.7 MAL-PEG_2_-NHS release in response to AC magnetic field from layer-by-layer film on magnetic nanoparticles

The MAL-PEG_2_-NHS release from LbL film on magnetic nanoparticles (Fe_3_O_4_) was conducted following the article (Fujisawa et al., 2024). A solution of poly (SS-*co*-FMA) (4,900 µL)—a polyanion adjusted to a concentration of 5.0 mg mL^-1^ in Milli-Q water containing 150 mM NaCl—was added to a 10 mL sample tube. Subsequently, 100 µL of an MNP dispersion was added to the poly (SS-*co*-FMA) solution and stirred for 15 min. A sample tube containing the MNP/poly (SS-*co*-FMA) solution was placed on a neodymium magnet for 10 min, and the supernatant (1.0 mL) was placed in a 1.5 mL Eppendorf tube. To wash the polymer-coated MNPs, the aliquoted MNP dispersion was separated in a centrifuge pre-cooled to 4°C at 15,000 rpm for 15 min, and the supernatant was removed. Fresh 1.0 mL Milli-Q water was added to the polymer-coated MNPs and pipetted. The particles were separated via centrifugation and cooled to 4 °C at 15,000 rpm for 15 min. These washing process was repeated twice to obtain MNPs with a single layer of poly (SS-*co*-FMA). The PAH solution (a polycation) was then prepared in Milli-Q water at a concentration of 5 mg mL^-1^ with 150 mM NaCl. PAH solution (1.0 mL) was added to the MNPs coated with poly (SS-*co*-FMA) and stirred for 15 min. The sample tube containing the MNP/poly (SS-*co*-FMA) solution was placed on a neodymium magnet for 10 min, and the 1.0 mL of supernatant was placed in a 1.5 mL Eppendorf tube. The aliquoted MNP dispersion was separated in a centrifuge pre-cooled to 4 °C at 15,000 rpm for 15 min. To wash the polymer-coated MNPs, the supernatant was removed, and fresh 1.0 mL Milli-Q water was added to the polymer-coated MNPs and pipetted. The particles were separated via centrifugation and cooled to 4 °C at 15,000 rpm for 15 min. The washing process with Milli-Q water was repeated twice to obtain MNPs with PAHs stacked on the poly (SS-*co*-FMA) layer. By repeating these processes an arbitrary number of times, MNPs with up to eight polymer layers were obtained. Hydrodynamic diameter measurements were performed using an ELSZ-2000 instrument (Otsuka Electronics Co., Ltd., Osaka, Japan). A high-power semiconductor laser was used as the incident beam. After the concentration of each polymer-coated MNP was adjusted to a level in the measurable range with Milli-Q water, at each layer number, filtered by a 0.45 μm pore size, 13 mm-diameter polytetrafluoroethylene syringe filters (Membrane Solutions, LLC, WA, USA) then added the solution in disposable cuvettes were used for the particle size measurements at 25°C.

To evaluate the MAL-PEG_2_-NHS release from MNPs, the AC magnetic field irradiation time was fixed, and the amount of MAL-PEG_2_-NHS released was evaluated. MAL-PEG_2_-NHS was dissolved in 1.0 mL at a concentration of 5.0 mg mL^-1^ of an MNPs solution with up to eight layers of polymer and bound via the DA reaction overnight. To remove the unbound MAL-PEG_2_-NHS, the MNPs solution was centrifuged and washed with 4.0 mL of Milli-Q water, excluding the supernatant. The washing process was repeated three times. Subsequently, 1.0 mL of the MNPs solution was placed in a 1.5 mL microtube such that the sample was at the center of the coil of the AC magnetic field apparatus and was subjected to an AC magnetic field using HOSHOT2 at 283 kHz and 184 A for 15 min. The samples were then cooled in a coil using water. The supernatant containing the released MAL-PEG_2_-NHS was collected. To calculate the concentration of released MAL-PEG_2_-NHS, the ultraviolet–visible (UV–vis) absorption of the MAL-PEG_2_-NHS in 10 µL of the collected supernatant was measured using a NanoDrop OneC (Thermo Fisher Scientific K.K., MA, USA). The concentration of the released MAL-PEG_2_-NHS was calculated from the calibration curve of MAL-PEG_2_-NHS dissolved in Milli-Q water at various concentrations. A minimum of three measurements were performed for each sample.

## 4 Conclusion

In summary, we successfully formed an LbL-based reaction environment for the DA reaction using FMA copolymers. The Py-value, which indicates the degree of polarity, increased when the number of polymer layers was 40 compared to 8, indicating that the polarity of the reaction environment could be controlled while using the same polymer by simply increasing the number of layers. Notably, we succeeded in promoting the binding of DA MAL-PEG_2_-NHS in the layers more effectively than the binding achieved in the typical solvent system. To the best of our knowledge, this is the first report on the construction of an enhanced reaction environment for the DA reaction on the surface of a substrate. This technology can pave the way for the industrialization of dynamic covalent chemistry, as the reaction rate can be accelerated without changing the chemical structures of the substrates. Furthermore, revere reaction (rDA) was successfully triggered by AC magnetic field irradiation by employing the LbL-coating on MNPs.

## Data Availability

The raw data supporting the conclusions of this article will be made available by the authors, without undue reservation.
