# Supplementary material for: Layer-by-layer assembled film can serve as an enhanced reaction environment for Diels–Alder reaction
Source: Front Chem. 2024 Dec 12;12:1524096. doi: 10.3389/fchem.2024.1524096 (PMC11672198; doi:10.3389/fchem.2024.1524096)
Supplement: Supplementary file 1 [file DataSheet1.docx]

Supplementary Material

Layer-by-Layer Assembled Film Can Serve as an Enhanced Reaction Field for Diels–Alder Reaction

Nanami Fujisawa^1,2^, Mitsuhiro Ebara^1,2,3*^

^1^Research Center for Macromolecules and Biomaterials, National Institute for Materials Science (NIMS), 1-1 Namiki, Tsukuba, Ibaraki 305-0044, Japan

^2^Graduate School of Pure and Applied Sciences, University of Tsukuba, Tsukuba, Ibaraki 305-0006, Japan

^3^Department of Materials Science and Technology, Tokyo University of Science, Tokyo 125-8585, Japan

Corresponding Author

*Mitsuhiro Ebara; E-mail: EBARA.Mitsuhiro@nims.go.jp

 
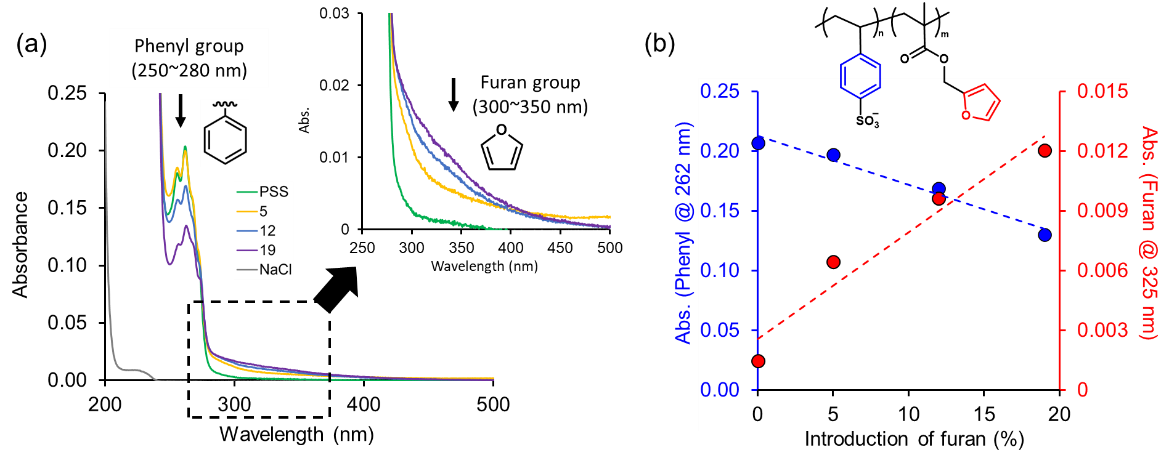


Figure S1. Physical properties of the synthesized polymers (a) Change in UV-vis absorption at each FMA incorporation ratio (b) Change in phenyl group and furan group incorporation ratio.


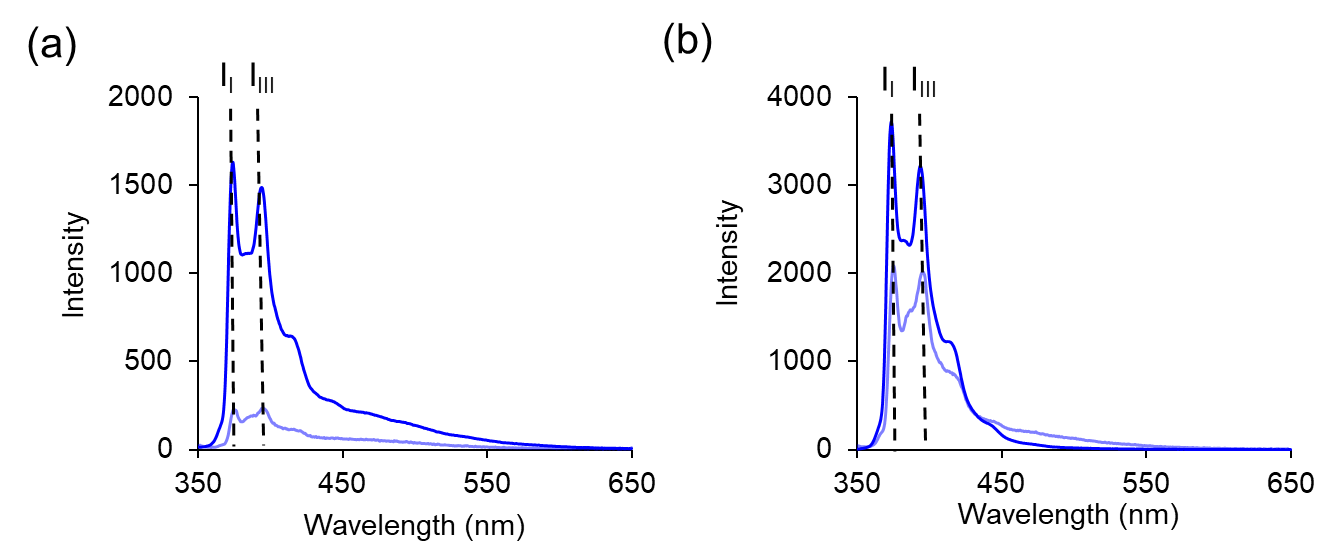


Figure S2. Fluorescence intensity of pyrene in (a) 8 layers (b) 40 layers. Blue indicates fluorescence intensity with the membrane immersed in MiliQ water; light blue indicates fluorescence intensity in the dry state.
